# The Effects of Loaded Plyometric Exercise during Warm-Up on Subsequent Sprint Performance in Collegiate Track Athletes: A Randomized Trial

**DOI:** 10.3390/sports8070101

**Published:** 2020-07-17

**Authors:** Kalin A. Tomlinson, Ken Hansen, Daniel Helzer, Zakkoyya H. Lewis, Whitney D. Leyva, Meghan McCauley, William Pritchard, Emma Silvestri, Monica Quila, Michael Yi, Edward Jo

**Affiliations:** Human Performance Research Laboratory, Department of Kinesiology and Health Promotion, California State Polytechnic University, Pomona, CA 91768, USA; katomlinson@cpp.edu (K.A.T.); kahansen@cpp.edu (K.H.); dphelzer@cpp.edu (D.H.); zakkoyyal@cpp.edu (Z.H.L.); whitleyva@gmail.com (W.D.L.); mmmccauley@cpp.edu (M.M.); william.pritchard990@gmail.com (W.P.); emmasilvestri7@gmail.com (E.S.); maquila@cpp.edu (M.Q.); myi@cpp.edu (M.Y.)

**Keywords:** post-activation potentiation, sprinting, plyometric exercise, track and field

## Abstract

Prior evidence demonstrates the efficacy by which plyometric activities during warm-up conditions augment the subsequent performance in power-centric exercise. We investigated the acute effects of loaded jump squats incorporated into a standard sprinters’ warm-up protocol on subsequent sprint performance in collegiate track athletes. Sprint times of 22 male and female collegiate track athletes were measured in 10-m intervals during a 30-m sprint trial following a standard sprinters’ warm-up routine with or without plyometric exercise. Subjects were tested on two separate occasions, once with loaded jump squats as the experimental treatment (two sets of eight jumps, load = 13% bodyweight) (PLYO) and once with time-equated rest as the control treatment (CON). Treatments were implemented following a standard sprinters’ warm-up routine familiar to the subjects. A dependent T-test was used for comparison of sprint interval times between conditions with a significant effect indicated by a *p*-value < 0.05. Sprint time did not differ between CON vs. PLYO at the 10 m (PLYO = 1.90 ± 0.12 s vs. CON = 1.90 ± 0.11 s, *p* = 0.66), 20 m (PLYO = 3.16 ± 0.21 s vs. CON = 3.15 ± 0.19 s, *p* = 0.53), and 30 m (PLYO = 4.32 ± 0.32 s vs. CON = 4.31 ± 0.28 s, *p* = 0.61) intervals. There was no interaction between treatment and sex, sex-specific ranking (above vs. below sex-specific mean), or sprint event (short vs. short–long vs. long) for 10 m, 20 m, or 30-m interval sprint times. At least within the limits of the current investigation, no evidence was provided to suggest that jump squats loaded at 13% bodyweight are an effective means to acutely potentiate sprint performance in collegiate track athletes. However, a further examination of responders indicates that the present loaded jump squat protocol may preferentially potentiate sprint performance in faster male athletes.

## 1. Introduction

An ongoing debate in sprint events in track and field is centered around the optimum pre-competition warm-up protocol. Although the warm-up is largely viewed as a general routine for pre-competition preparation, a physiological phenomenon referred to as post-activation performance enhancement (PAPE) enables an alternate perspective of the warm-up as a method for acute performance augmentation. Post-activation performance enhancement, classically referred to as post-activation potentiation (PAP), is a recent term used to describe an acute increase in voluntary muscular performance in response to an immediate voluntary contractile history that is muscle-specific and high-intensity in nature [1,2]. The nuances between PAPE and PAP have become progressively clearer in that there are multiple mechanisms underlying PAPE, one of which includes PAP, along with increased muscle temperature, motor unit activation, and myofiber water content [1]. PAP specifically refers to an enhanced maximal twitch force moments after intense voluntary contractions and is explained by improved myofilament interactions mediated by increased calcium influx and myosin light chain phosphorylation [3,4,5]. This may or may not explain incidences of PAPE, since again, it is likely underpinned by multiple factors. Often, observations of PAPE in practical or research settings are unaccompanied by measurements of PAP and thus, in such a case, PAPE would be the appropriate terminological description.

The mode of exercise used with the intention of acutely potentiating performance is often referred to as a preload activity and has shown to include moderate to heavy resistance or high-velocity/-power exercises [6,7,8,9,10,11]. Moreover, due to the transient nature of PAPE, effects would only manifest in brief maximum-effort exercise events, such as those involved in track and field (e.g., single sprint, vertical jump, long-jump, etc.). Blazevich and Babault [1] stated that there is equivocal evidence regarding the proper conditions required to elicit a PAPE response, which significantly limits its practical application in sports. It is not a question of whether the PAPE phenomenon exists, but rather how it can be systematically exploited by athletes. Past research outcomes are generally mixed with regards to the optimum preload activity conditions due to the number of interwoven factors that have shown to influence whether a PAPE response can be successfully achieved. These mainly include training history, familiarity to the preload activity or the exercise intended to be enhanced and the characteristics of preload activity (i.e., intensity, volume, mode).

For lower body PAPE responses, moderate to heavy squats with relative loads ranging between 20–90% of one repetition maximum (RM) have demonstrated variable degrees of efficacy in potentiating sprint and jump performances especially in athletes or well-trained subjects familiar to loaded squat exercise [10,11,12,13]. For instance, Chatzopoulos et al. [13] showed in resistance-trained, recreational athletes, that 30-m sprint performance was enhanced 5 min following 10 non-continuous repetitions of half squats at 90% 1RM. In a more homogenous subject pool of Rugby Union players, three repetition of the back squat at 90% 1RM resulted in a faster sprint time within 4 min [12]. Despite such evidence supporting the use of heavy back squats to acutely enhance sprint performance, it remains highly impractical in a competitive race scenario, such as in track and field. Besides the need for heavy equipment, there is the inherent possibility or potential fear of fatigue and/or injury associated with relatively heavy exercise. Thus, researchers have explored more pragmatic methods that could be incorporated into a standard pre-competition warm-up routine with relative ease, such as sub-maximally loaded plyometrics [8]. However, the question of efficacy remains in doubt; although, the emergence of recent data may provide some limited evidential support.

In a prior investigation, Turner et al. [14], found that three sets of 10 repetitions of alternate leg bounding (plyometric exercise) produced an enhancement in 10-m and 20-m sprint acceleration compared to a walking warm-up control. Similarly, Creekmur et al. [8] recently reported a reduced 20-m and 40-m sprint time following a plyometric warm-up protocol consisting of weighted jump squats using a 11.3 kg plate specifically in sprint athletes. A unique aspect of their experimental approach was that the plyometric preload treatment was incorporated into a standard pre-race warm-up routine for competitive sprinters. Based on these limited findings, there appears to be some merit for the use of plyometric exercise prior to sprint events, especially considering its practicality as a component of a typical on-field warm-up routine.

PAPE (or classic “PAP”) has been a topic of debate in the sports science community for a relatively long time. There are numerous mechanistic studies supporting the presence of the PAPE and PAP phenomenon, however, there are fewer studies demonstrating the efficacy of practical preload activities especially for competition settings (i.e., track and field). As described above, there is evidence, albeit limited, supporting the use of a plyometric-based preload activity. However, the paucity of applicable data precludes the systematic implementation of plyometric exercise in pre-race warm-up situations for the purpose of PAPE especially for competitive sprint athletes. Limitations of Creekmur et al. [8] included the use of a constant 11.3-kg load for all subjects and the inclusion of a small sample of male athletes. Therefore, practical inferences from this data remain limited and thus, furthering this line of research inquiry is warranted to generate more substantial and applicable conclusions. It would be worthwhile to examine a plyometric preload activity using relative loads incorporated into a standard sprinters’ warm-up routine. Moreover, the inclusion of competitive male and female sprinters of a single-track team who have relatively similar training histories and performance demands would improve the control of the experiment and interpretation of data. Altogether, a study incorporating these factors would conceivably improve the current understanding of the application of practical preload activities in real-life sporting situations in which PAPE would be useful, i.e., sprint events. Thus, the aim of this study was to investigate the acute effects of loaded jump squats incorporated into a standard sprinters’ warm-up protocol on subsequent sprint performance in collegiate track and field athletes. It was hypothesized that sprint performance, as measured by 10-m, 20-m, and 30-m sprint interval times, would be acutely enhanced by the inclusion of a loaded jump squat preload activity.

## 2. Materials and Methods

### 2.1. Experimental Design

**Figure 1:**
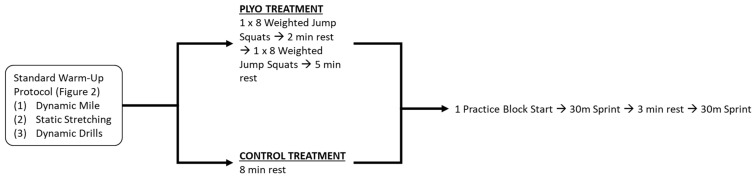
Schematic of Experimental Protocol.

A randomized, crossover experimental design was implemented for this study on National Collegiate Athletic Association (NCAA) Division II student athletes from California State Polytechnic University, Pomona (CPP) (Figure 1). Subjects visited the laboratory and the campus track at CPP on two separate occasions, separated by 7 days. Each subject underwent protocols administered by the same investigators for both visits. During the first visit, subjects underwent basic descriptive anthropometric measures and body composition assessment via multi-frequency bioelectrical impedance analysis (BIA) using InBody 770 (Biospace, CO. Ltd., Seoul, South Korea). During Visit 1, subjects performed one of two pre-sprint trial protocols, (1) control treatment (CON) (standard sprinter-specific warm-up then passive rest) or (2) experimental treatment (PLYO) (standard sprinter-specific warm-up then loaded plyometric jump squat exercise). During Visit 2, subjects received the alternate treatment. Both treatment protocols are described in detail below. The order of treatment was randomized by a single investigator via digital coin-flip (random.org) and counterbalanced. Following the PLYO and CON treatments, subjects performed 2 trials of a 30-m sprint on an official NCAA-approved 400-m track on a straight 100-m stretch. Laser timing gates were placed at the 10-m, 20-m, and 30-m marks along the track. Data from the 1st, 2nd, and best trial as well as the average sprint times of both trials were analyzed individually. Similar outcomes were produced across each analysis, therefore, the results from the 1st trial are presented here as this would be the most practical from a real-life competition perspective. Although the 100-m dash is a common event for sprint athletes, the 30-m distance for assessment was selected for the following reasons: (1) subjects included track athletes who participate in a variety of sprint events other than the 100-m dash (e.g., 100–400-m dash, short distance hurdles, heptathlon), (2) the 30-m sprint test is commonly administered to assess performance in track and field sprint athletes and (3) subjects were required to perform 2 consecutive sprint trials and thus, a 30-m distance was selected partially to minimize the influence of fatigue. Subjects were asked to refrain from heavy exercise a day prior and the day of their visit. Moreover, dietary intake was recorded the day before and day of their first visit. Subjects were required to replicate their dietary intake, hydration, and sleep period (monitored via self-recorded logs) prior to their second visit.

### 2.2. Subjects

Twenty-two healthy, college-aged, male (*n* = 12, age = 19.8 ± 1.5, bodyweight = 71.3 ± 8.5 kg, height = 176.9 ± 5.2 cm) and female (*n* = 10, age = 19.1 ± 1.3y, bodyweight = 55.3 ± 4.4 kg, height = 168.8 ± 2.1 cm) NCAA Division II athletes of the Track and Field team at CPP were recruited for this study. Prior to participation, each subject completed a pre-participation exercise and health history questionnaire and signed a document of informed consent. Subjects met the following inclusion criteria: (1) age = 18 to 32 years, (2) a competitive athlete currently on the CPP Track and Field team roster competing in sprint events (e.g., 100–400 m, short distance hurdles, etc.) for at least one collegiate season, and (3) eligible for competition, practice, and training according to NCAA regulations. Subjects were excluded from participation if they reported a medical or surgical history that would contraindicate the experimental protocol and/or confound the interpretation of results. These included but were not limited to: (1) cardiovascular, pulmonary, metabolic, or renal diseases, (2) hypertension, (3) smoking, and (4) use of any medication, including those with cardiovascular, pulmonary, hyperlipidemic, hypoglycemic, or hypertensive effects. In addition, subjects were excluded if they utilized dietary ergogenic aids daily within 6 weeks prior to the study. Caffeine intake of 6 mg/kg bodyweight/day called for exclusion. Daily use of nutritive supplements (e.g., whey protein or multivitamins) did not call for exclusion. This study was approved by the Institutional Review Board and subjects were informed of the benefits and risks of the investigation prior to signing an institutionally approved informed consent document to participate in the study.

### 2.3. Procedures

#### 2.3.1. Experimental and Control Treatments

**Figure 2:**
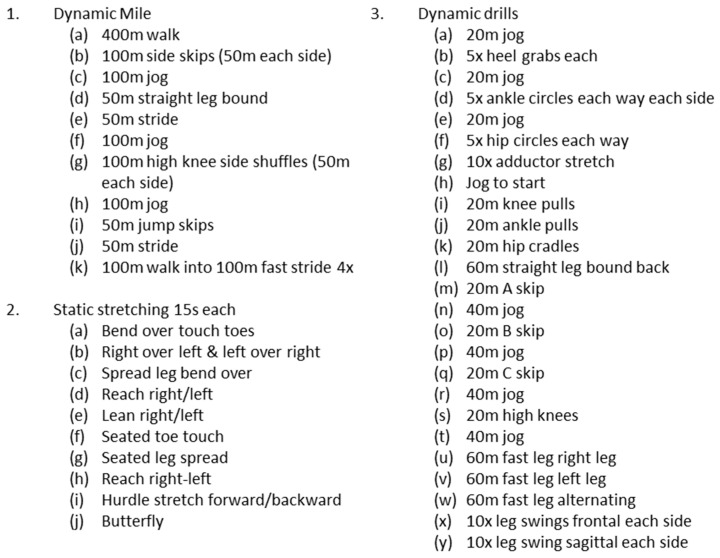
Description of Standardized Sprinter Warm-Up Protocol.

Figure 1 displays a diagram of the protocol for both CON and PLYO treatments. Prior to each treatment, subjects underwent a standardized warm-up protocol as described in detail in Figure 2. This warm-up protocol was implemented because it is the same routine used by each subject prior to their competitions. After the standardized warm-up, the PLYO treatment included 1 set of 8 loaded squat jumps using a vest containing a load equal to 13% of the subject’s total bodyweight; after a 2 min rest period, subjects performed another set of 8 jumps followed by a 5 min rest period. During the 5 min rest period, subjects put on track footwear/spikes and adjusted their blocks according to their own specifications. A loading protocol of 13% bodyweight was selected on the basis of Creekmur et al. [8] who indicated that the absolute load of 11.3 kg equated to the lower range of 13% of body mass of their subject pool. For the CON treatment, subjects passively rested for 8 min after the standardized warm-up (time equated to PLYO treatment). During this time, subjects put on their spikes and adjusted their blocks to their specifications. Block adjustments for each subject was matched between CON and PLYO treatments. After the treatment protocol, subjects performed one practice block start followed by the 30-m sprint trials.

#### 2.3.2. Thirty-Meter Sprint Test

A laser timing gate system (Zybek Sports, Power Dash Timing System, Westminister, CO, USA) was utilized to measure 30-m sprint time and 10-m and 20-m interval times (ICC = 0.91). The timing gates were placed at 10-m, 20-m, and 30-m distances along an official 100-m track at CPP. A tape measure was used to mark each distance from the starting line on the 100-m stretch of the track. The markers remained untouched between the visits; however, distances were reconfirmed prior to the second visit. Subjects began the test in a block start position set to their individual preference. Subjects began the sprint trial at their own volition without any visual or auditory cue. Timing was initiated when the subject’s hands placed in between the first laser emitter/sensor and reflector were lifted off the ground instantaneously triggering the timer. Each subject was provided 2 trials separated by 3 min of rest. Data from the first trial is presented here as explained above.

### 2.4. Statistical Analyses

Using a Shapiro Wilk test and a confidence interval of 95% it was confirmed that the dependent variables had a normal distribution. A paired samples t-test was used to compare CON vs. PLYO on 10-m, 20-m, and 30-m sprint interval times. The primary outcome for this study was the overall group assessment of sprint time for 10-m, 20-m and 30-m distances. The secondary outcome was an assessment of sprint times based on subgrouping variables which included sex, sex-specific sprint ranking (above vs. below sex-specific 30-m sprint time mean), and sprint event (short vs. short–long vs. long). For subgroup analyses, a general linear model was used to test for interactions of treatment x sex, sex-specific sprint ranking, and sprint event. Subjects were analyzed according to their individual sprint event as categorized by short (subjects who compete only in 100 and/or 200 m), short–long (subjects who compete in 200 and 400 m) and long (subjects who compete in 400 m) since training background may differ slightly depending on the sprint event in which the athlete competes in. Analyses were performed using SPSS 23 (IBM, Armonk, NY, USA) with significance set at *p* < 0.05. Effect sizes (ES) for sprint time were calculated by Cohen’s d (mean of CON—mean of PLYO/pooled standard deviation). The ES interpretation were as follows: −0.8 = large decrease, −0.5 = moderate decrease, −0.2 = small decrease, −0.19–0.19 = trivial, 0.2 = small increase, 0.5 = moderate increase, 0.8 = large increase (a negative ES represents a faster sprint during PLYO vs. CON, and vice versa for a positive ES) [15]. A simple linear regression was performed to analyze the relationship between control 30-m sprint time and 30-m sprint time difference score (PLYO-CON) to examine whether baseline sprint time impacts whether one experiences PAPE. Sprint time data are presented as mean ± standard deviation (SD). Sample size determination was limited by the available sprint athletes of the CPP Track and Field team willing to volunteer.

## 3. Results

Table 1 and Table 2 provide mean data for sprint interval times and effect sizes, respectively, for the whole study cohort and sub-groups. Sprint time did not differ between CON vs. PLYO at the 10 m (*p* = 0.66), 20 m (*p* = 0.53), and 30 m (*p* = 0.61) intervals. There was no treatment x sex interaction for 10 m (*p* = 0.19), 20 m (*p* = 0.25), or 30 m (*p* = 0.16) interval sprint times. There was no treatment x sex-specific sprint time ranking interaction for 10 m (*p* = 0.79), 20 m (*p* = 0.99), or 30 m (*p* = 0.83) interval sprint times. There was no treatment x sprint event interaction for 10 m (*p* = 0.70), 20 m (*p* = 0.60), or 30 m (*p* = 0.33) interval sprint times. There was no significant correlation between the difference score in sprint time between PLYO and CON (PLYO-CON = difference score) vs. 10-m, 20-m, or 30-m CON sprint time. Thus, PAPE responses were not related to how slow or fast the subject was.

## 4. Discussion

The objective of this study was to investigate the effects of an additional loaded plyometric warm-up activity to a standard sprinters’ warm-up protocol on subsequent sprint performance in collegiate track athletes. It was hypothesized that sprint performance would be potentiated by the inclusion of loaded jump squats compared to the standard sprinter-specific warm-up protocol with time-matched passive rest. As an executive summary of the findings, two sets of eight jump squats using a load 13% of bodyweight performed after a standard warm-up routine failed to potentiate sprint performance as determined by 10-m, 20-m, and 30-m interval sprint times. Thus, the present hypothesis was rejected. Secondary findings included no interaction between sex, sex-specific performance ranking, or type of sprint event in which the subject competed. However, characteristics of responders (PAPE observed) vs. non-responders (PAPE not observed) will be discussed below.

**Figure 3:**
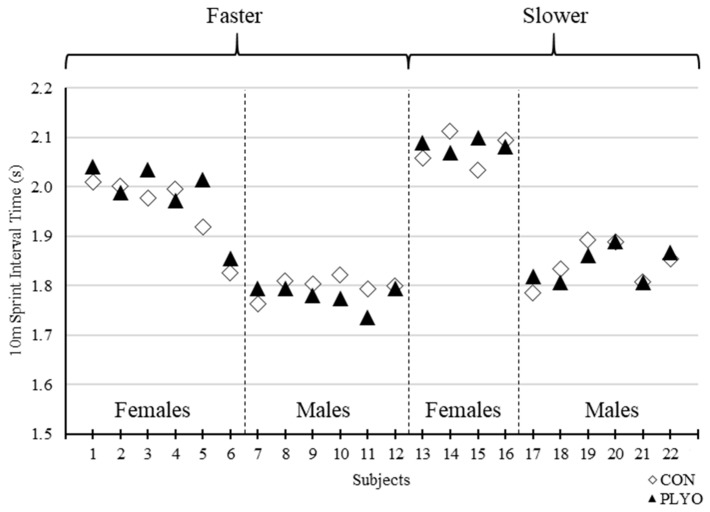
Individual 10-m sprint interval times for control (CON) and plyometric (PLYO) treatments grouped by sex and 30-m performance ranking. “Faster” and “Slower” = above and below sex-specific mean for control 30-m sprint time, respectively.

**Figure 4:**
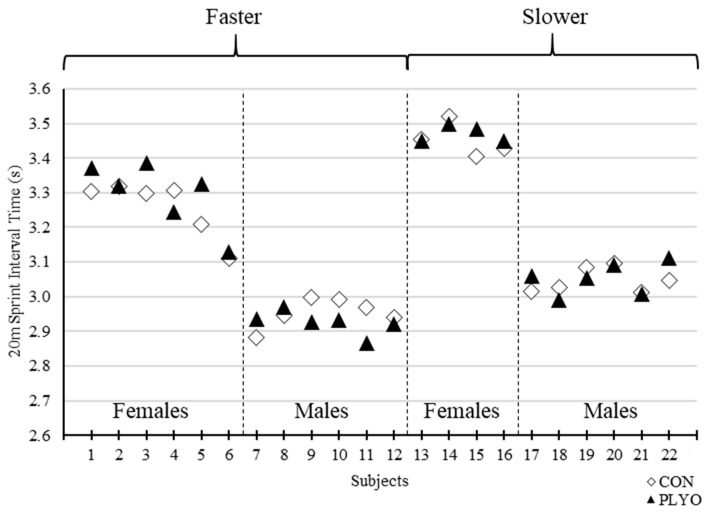
Individual 20-m sprint interval times for control (CON) and plyometric (PLYO) treatments grouped by sex and 30 m-performance ranking. “Faster” and “Slower” = above and below sex-specific mean for control 30-m sprint time, respectively.

**Figure 5:**
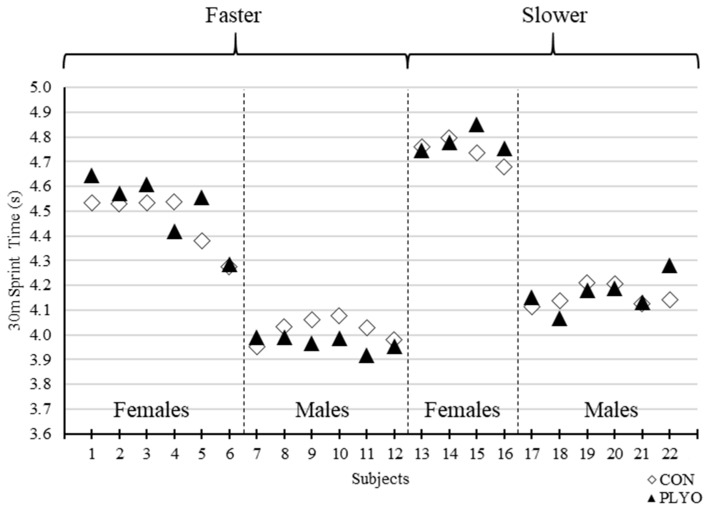
Individual 30-m sprint interval times for control (CON) and plyometric (PLYO) treatments grouped by sex and 30 m-performance ranking. “Faster” and “Slower” = above and below sex-specific mean for control 30-m sprint time, respectively.

The current findings conflict with prior studies of similar inquiry particularly Creekmur et al. [8] who also examined the effects of loaded jump squats on subsequent sprint performance but in solely male collegiate track athletes. Creekmur et al. [8] utilized a 40-m sprint test with assessment of 20-m interval time and found that performance was significantly improved when subjects incorporated two sets of eight jump squats using an absolute load of 11.3 kg (12.8 – 16.6% of bodyweight). Creekmur et al. [8] showed an average 0.04 s faster 20-m sprint time compared to the control condition while the current results showed a mean, non-significant, 0.01 s faster 20-m sprint time vs. control. Moreover, the male subgroup within the current study (*n* = 12) also demonstrated a non-significant 0.01 s faster sprint time than control and thus, there appears to be clear discrepancies in the outcomes between the two studies. Like Creekmur et al. [8], the present investigation utilized a relatively homogenous subject pool of collegiate track athletes familiar to sprinting. However, our analysis comprised of a wider spectrum of track athletes that included females and more divergent 30-m sprint times across the entire cohort. This may explain the opposing results between the two studies as Creekmur et al. [8] incorporated a more homogenous group of male Division I collegiate track athletes. When examining data specific to sex or performance ranking, no PAPE effects were detected in the present study. What may corroborate the findings of Creekmur et al. [8], however, is an examination of responders or individual subjects who demonstrated PAPE. The subgroup exhibiting the greatest number of responders (45% of all responders) and average improvement in sprint times during PLYO vs. CON were male subjects with 30-m control sprint times less than the male-specific mean of 4.09 s (i.e., the “faster male” subgroup) (Figure 3, Figure 4 and Figure 5). The faster male subjects exhibited 0.05 s and 0.03 s faster 30 m (CON = 4.02 ± 0.05 s vs. PLYO = 3.97 ± 0.03 s) and 20 m (CON = 2.95 ± 0.04 s vs. PLYO = 2.92 ± 0.04 s) split times, respectively, during the PLYO condition. This subgroup is comparable in performance characteristics to the study cohort of Creekmur et al. [8] and interestingly demonstrated similar outcomes. Creekmur et al. [8] reported a mean control 20-m sprint time of 3.17 ± 0.09 s and a potentiation of 0.04 s following the preload activity. Therefore, loaded jump squats incorporated into a standard sprinter’s warm-up routine may be preferentially effective as a PAPE stimulus for faster male sprinters. Although sex-specific responses to a given preload activity have not been demonstrated previously, it was worthwhile to include female athletes in the present investigation as prior PAPE studies differed in athlete population, preload activity, and outcome measures and cannot be universally extrapolated to all PAPE situations. Although our analysis failed to demonstrate any interaction of sex, individual subject data may hint to some influence of sex and/or ranking in conditions specific to those presented in the current study. This data may be useful to guide future research of similar inquiry and general design. Regardless, the evidence presented herein does not fully substantiate interactions of sex and/or ranking on the effects of the preload activity. To note, Creekmur et al. [8] analyzed averaged data across two sprint trials while the present study used the data from the first sprint trial. A secondary set of analysis using the average sprint time data did not alter the overall results (data not presented).

Like most studies of similar inquiry, there are those who respond and those that do not. A greater challenge in human performance PAPE research is delineating the common characteristics or factors among responders to a given preload activity under specific conditions. In relation to the current investigation, it may be worthwhile to reexamine the higher-ranking male sprinters to assess the reliability of the current preload activity in eliciting a PAPE effect. In the case that the preload activity does elicit a consistent PAPE response in this particular subgroup of responders, a follow-up investigation with perhaps replication of the current study design with a larger sample size for subgroup analyses would determine whether the PLYO treatment preferentially potentiates faster male sprinters. Moreover, it must be noted that an important limitation in the present study involves the lack of confirmation on whether peak sprint speed was achieved within the 30-m distance. Questions remain whether a greater sprint distance would reveal differences between treatments. This limitation may also explain the discrepant results between Creekmur et al. and the current study. There is no question however, that the complexity surrounding PAPE applications is the interrelationship between multiple factors that determines whether a consistent potentiation effect can be achieved.

**Figure 6:**
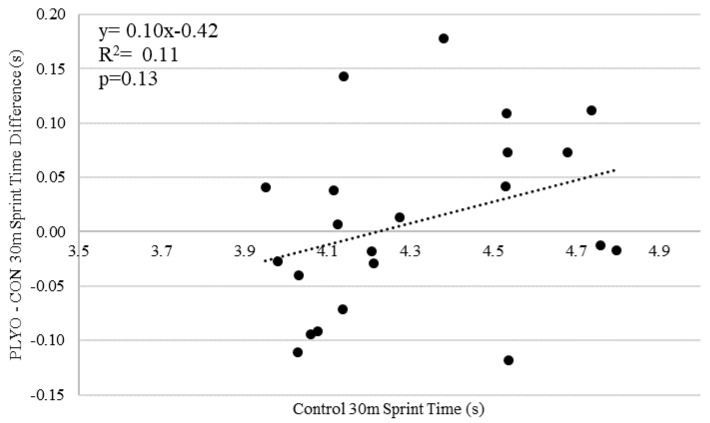
Relationship between 30-m sprint time difference score (PLYO-CON) and Control 30-m sprint time. A negative difference score reflects a potentiating effect.

One of the major factors that has shown to influence the effectiveness of a given preload activity is training status/history and performance capacity. In a previous study of by Jo et al. [10], results showed that stronger subjects (above average relative back squat) demonstrated PAPE during a subsequent cycling sprint test compared to the weaker subjects. For the current study, the inclusion of well-trained collegiate sprint athletes was determined on the basis of previous research such as Jo et al. [10] and Gourgoulis et al. [11] in which training status or history influenced the efficacy of a given preload activity. For instance, when the strongest or more well-trained subjects were isolated from the overall group analysis, vertical jump potentiation increased from a 2.39% to a 4.01% improvement compared to control. It appears from the present study that there might be further intricate training status factors that influence the presence or lack of potentiation. About half of the current subjects potentiated, and as mentioned above, a majority were faster male sprinters as shown in Figure 6. However, our mixed factorial and correlation analyses failed to detect any significant interaction or relationship, respectively, of any grouping factors such as sex, sex-specific ranking, and sprint event type. Thus, the present study was unable to elucidate potential confounders, rather, a general description of those subgroups who demonstrated sprint potentiation was provided. As discussed earlier, future PAPE research specifically in the sprint athlete population should focus on whether the potentiation effects of a given preload activity, such as jump squats loaded with 13% of bodyweight, is preferential to a particular type of athlete.

To gain at least some practical inferences from the current data, individual data of 3 female subjects and 8 male subjects showed potentiation effects with an average drop in sprint time of 0.07 s. At first glance, this difference may not appear as a vast margin of difference, but when placed under competition scenarios, this improvement in time may certainly dictate placement. When examining the results for the men’s 100-m sprint final at the 2019 Division II National Championship for the NCAA, the difference between first and second place was 0.05 s. Thus, the responders of the current investigation may have moved up by at least one place during competition. Indeed, evidence-based practices is fundamentally predicated on the probabilities of an effect, but at least in the context of performance potentiation, individualized effects and feedback in response to a preload activity must be considered. We anticipate with future exploration, further development of systematic approaches to sprint potentiation will be possible.

The application of PAPE in sports is arguably most relevant to athletes who compete in sports comprised of individual events particularly requiring maximum muscular power output across short distances, like a 100-m sprint, Olympic lifting, powerlifting, etc. Due to the transient nature of PAPE, a preload activity may not be suitable for most team sport athletes such as in soccer and basketball since it is likely that any potentiating effects would dissipate relatively quickly having no influence on overall game performance. However, specific situations within team sport competition may benefit from an effective preload activity. For example, in American football, a field goal attempt, more specifically, kicking power, may be acutely enhanced in response to a preload activity performed moments prior on the sidelines. Additional areas in sports in which PAPE may be relevant is during performance testing situations, e.g., combines, which generally comprise of assessments such as lower and upper body strength, sprint performance, and vertical jump. As for the type of preload activity, a loaded jump squat that is relatively low in volume and intensity would be ideal if consistently effective because of the relative ease in which it can be incorporated into a pre-competition routine. However, as mentioned above, further research is needed to ascertain who would most benefit from the present preload activity. Adding to the existing complexity of PAPE elicitation, the characteristics of the preload activity may also influence whether one potentiates or not. Using Creekmur et al. [8] as a guideline, the current study tailored the loaded squat jumps to individual bodyweight (13% of bodyweight). As indicated earlier, Creekmur et al. [8] included 10 male sprint athletes and showed a potentiation effect following a similar loaded jump squat preload activity using an absolute load of 11.3 kg which was between 12.8% and 16.6% of their subjects’ bodyweight. The 13% relative loading protocol in present study was selected based on the lower end of this range. However, when examining the absolute load utilized by the present male sub-group (*n* = 12), approximately 9 kg was utilized during the preload activity which was lighter than the load implemented by Creekmur et al. [8]. Thus, the lack of potentiation within the male sub-group may be explained by inadequate loading. Moreover, the 11.3 kg load used by Creekmur et al. [8] was 14.8% of the mean bodyweight of the subject pool (76.3 kg), and thus, the relative load of 13% bodyweight used in the present study was potentially insufficient. In fact, when examining the entire subject pool (N = 22), the average absolute load was 8.3 kg, and only a single subject was prescribed a load comparable to that used by Creekmur et al. [8], i.e., 11.2 kg. Interestingly, this subject demonstrated a PAPE response. Although the present preload activity intensity was individualized, various relative or absolute intensities and perhaps volumes across a spectrum would perhaps induce different outcomes across subjects. In other words, some individuals may potentiate in response to a particular intensity (or volume) vs. another individual. For example, a bodyweight jump squat might be more effective in slower or less-trained athletes compared to loaded jump squats as demonstrated by Turner et al. [14]. The question also remains whether bodyweight is the most appropriate variable to normalize intensity for a loaded jump squat preload activity. Potentially, a jump squat load relative to a 1RM of a comparable exercise, such as the front squat, would be better suited. Moreover, further investigation of specificity of the preload activity may be warranted since to our knowledge, only vertical movements have been explored as a potential PAPE stimulus for sprinting. There is no question that the nature of an effective preload activity remains a perplexing issue in PAPE research and application.

In conclusion, the addition of loaded jump squats to a standard sprinters’ warm-up routine failed to demonstrate efficacy in acutely enhancing subsequent sprint performance in collegiate sprint athletes. Of the responders, a majority were faster males, and thus, future inquiry to elucidate the effects of the current preload activity in this specific population would be especially insightful. Furthermore, greater emphasis on subject pool homogeneity in human performance PAPE research especially as it relates to training history would afford a clearer picture on the efficacy of a given preload activity in a population-specific manner. At least within the limits of the current investigation, no evidence is provided to suggest that jump squat exercise loaded at 13% bodyweight following a standard warm-up routine is an effective means to acutely enhance sprint performance in collegiate track athletes.

## 5. Practical Application

PAPE responses to the present preload activity appear to be highly individualized and possibly preferential to higher-ranking male sprinters. The addition of loaded jump squats into a standard pre-competition warm-up routine, although practical, should be implemented with caution as some may experience an acute detriment to subsequent sprint performance. Until further research can substantiate the specific population of sprint athletes responsive to the present preload activity, it is not recommended to universally apply these methods. However, athletes or coaches may assess the efficacy of the present preload activity on an individual basis and apply it during competition accordingly.

## Figures and Tables

**Table 1:** Thirty-meter sprint test interval times between experimental (PLYO) vs. control (CON) treatments.

Groupings	Intervals	PLYO (s)	CON (s)	Diff Score (s)	95% CI	*p*-value
Overall *n* = 22	10 m	1.90 ± 0.12	1.90 ± 0.11	−0.00 ± 0.04	−0.02–0.01	0.66
	20 m	3.16 ± 0.21	3.15 ± 0.19	−0.01 ± 0.06	−0.03–0.02	0.53
	30 m	4.32 ± 0.32	4.31 ± 0.28	−0.01 ± 0.08	−0.05–0.03	0.61
Male *n* = 12	10 m	1.81 ± 0.04	1.82 ± 0.04	0.01 ± 0.03	−0.01–0.03	0.20
	20 m	2.99 ± 0.08	3.00 ± 0.06	0.01 ± 0.05	−0.02–0.04	0.48
	30 m	4.07 ± 0.12	4.09 ± 0.08	0.02 ± 0.07	−0.02–0.07	0.33
Female *n* = 10	10 m	2.02 ± 0.07	2.00 ± 0.08	−0.02 ± 0.04	−0.05–0.01	0.16
	20 m	3.37 ± 0.12	3.34 ± 0.12	−0.03 ± 0.07	−0.07–0.01	0.12
	30 m	4.62 ± 0.17	4.58 ± 0.17	−0.05 ± 0.08	−0.10–0.01	0.12
Faster * *n* = 12	10 m	1.88 ± 0.12	1.88 ± 0.10	−0.00 ± 0.04	−0.03–0.02	0.72
	20 m	3.11 ± 0.21	3.11 ± 0.17	−0.00 ± 0.07	−0.05–0.04	0.81
	30 m	4.24 ± 0.30	4.24 ± 0.25	0.00 ± 0.09	−0.06–0.06	0.94
Slower * *n* = 10	10 m	1.94 ± 0.13	1.94 ± 0.13	−0.00 ± 0.03	−0.03–0.02	0.82
	20 m	3.22 ± 0.22	3.21 ± 0.21	−0.01 ± 0.04	−0.04–0.02	0.40
	30 m	4.41 ± 0.32	4.39 ± 0.31	−0.02 ± 0.07	−0.07–0.03	0.32
Short ^ *n* = 7	10 m	1.87 ± 0.15	1.88 ± 0.14	0.015 ± 0.03	−0.02–0.05	0.27
	20 m	3.09 ± 0.26	3.11 ± 0.26	0.02 ± 0.05	−0.03–0.07	0.31
	30 m	4.21 ± 0.38	4.25 ± 0.36	0.04 ± 0.05	−0.01–0.09	0.07
Short–Long ^ *n* = 10	10 m	1.97 ± 0.12	1.95 ± 0.10	−0.02 ± 0.05	−0.05–0.01	0.25
	20 m	3.26 ± 0.20	3.23 ± 0.17	−0.03 ± 0.06	−0.07–0.02	0.23
	30 m	4.46 ± 0.30	4.43 ± 0.25	−0.04 ± 0.09	−0.10–0.03	0.25
Long ^ *n* = 5	10 m	1.85 ± 0.03	1.85 ± 0.05	−0.00 ± 0.02	−0.03–0.03	0.80
	20 m	3.06 ± 0.04	3.05 ± 0.04	−0.02 ± 0.04	−0.06–0.03	0.44
	30 m	4.19 ± 0.06	4.16 ± 0.05	−0.03 ± 0.07	−0.11–0.06	0.41

* “Faster” and “Slower” = above and below sex-specific mean for 30-m sprint time, respectively. ^ Short = 100 m and 200 m; short–long = 200 m and 400 m; long = 400 m. CI = Confidence Intervals. Data presented as mean ± SD.

**Table 2:** Effect sizes (ES) and 95% confidence Intervals (CI) for sprint interval times.

Groupings	Intervals	Effect Size (d)	Interpretation	95% CI
Overall *n* = 22	10 m	0.00	Trivial	−0.13–0.13
	20 m	0.05	Trivial	−0.08–0.18
	30 m	0.03	Trivial	−0.09–0.16
Male *n* = 12	10 m	−0.25	Small Decrease	−0.48–−0.02
	20 m	−0.14	Trivial	−0.37–0.09
	30 m	−0.20	Small Decrease	−0.43–0.04
Female *n* = 10	10 m	0.27	Small Increase	−0.01–0.54
	20 m	0.25	Small Increase	−0.03–0.53
	30 m	0.24	Small Increase	−0.04–0.51
Faster * *n* = 12	10 m	0.00	Trivial	−0.23–0.23
	20 m	0.00	Trivial	−0.23–0.23
	30 m	0.00	Trivial	−0.23–0.23
Slower * *n* = 10	10 m	0.00	Trivial	−0.28–0.28
	20 m	0.05	Trivial	−0.23–0.32
	30 m	0.06	Trivial	−0.21–0.34
Short ^ *n* = 7	10 m	−0.07	Trivial	−0.47–0.33
	20 m	−0.08	Trivial	−0.47–0.32
	30 m	−0.11	Trivial	−0.50–0.29
Short–Long ^ *n* = 10	10 m	0.18	Trivial	−0.10–0.46
	20 m	0.16	Trivial	−0.12–0.44
	30 m	0.11	Trivial	−0.17–0.39
Long ^ *n* = 5	10 m	0.00	Trivial	−0.55–0.55
	20 m	0.25	Small Increase	−0.31–0.81
	30 m	0.54	Moderate Increase	−0.02–1.11

Positive ES = slower sprint time during experimental treatment (PLYO) vs. control treatment (CON), Negative ES = faster sprint time during PLYO vs. CON. * “Faster” and “Slower” = above and below sex-specific mean for control 30-m sprint time, respectively. ^ Short = 100 m and 200 m; short–long = 200 m and 400 m; long = 400 m.

## References

[1] Blazevich A.J., Babault N. (2019). Post-activation potentiation cersus post-activation performance enhancement in humans: Historical perspective, underlying mechanisms, and current issues. Front. Physiol..

[2] Cuenca-Fernández F., Smith I.C., Jordan M.J., MacIntosh B.R., López-Contreras G., Arellano R., Herzog W. (2017). Nonlocalized postactivation performance enhancement (PAPE) effects in trained athletes: A pilot study. Appl. Physiol. Nutr. Metab..

[3] Vandervoort A.A., Quinlan J., McComas A.J. (1983). Twitch potentiation after voluntary contraction. Exp. Neurol..

[4] Manning D.R., Stull J.T. (1979). Myosin light chain phosphorylation and phosphorylase A activity in rat extensor digitorum longus muscle. Biochem. Biophys. Res. Commun..

[5] Stull J.T., Manning D.R., High C.W., Blumenthal D.K. (1980). Phosphorylation of contractile proteins in heart and skeletal muscle. Fed. Proc..

[6] Hoffman J.R., Ratamess N.A., Faigenbaum A.D., Mangine G.T., Kang J. (2007). Effects of maximal squat exercise testing on vertical jump performance in american college football players. J. Sports Sci. Med..

[7] Gilbert G., Lees A. (2005). Changes in the force development characteristics of muscle following repeated maximum force and power exercise. Ergonomics.

[8] Creekmur C.C., Haworth J.L., Cox R.H., Walsh M.S. (2017). Effects of plyometrics performed during warm-up on 20 and 40 m sprint performance. J. Sports Med. Phys. Fit..

[9] French D.N., Kraemer W.J., Cooke C.B. (2003). Changes in dynamic exercise performance following a sequence of preconditioning isometric muscle actions. J. Strength. Cond. Res..

[10] Jo E., Judelson D.A., Brown L.E., Coburn J.W., Dabbs N.C. (2010). Influence of recovery duration after a potentiating stimulus on muscular power in recreationally trained individuals. J. Strength. Cond. Res..

[11] Gourgoulis V., Aggeloussis N., Kasimatis P., Mavromatis G., Garas A. (2003). Effect of a submaximal half-squats warm-up program on vertical jumping ability. J. Strength. Cond. Res..

[12] Duncan M.J., Thurgood G., Oxford S.W. (2014). Effect of heavy back squats on repeated sprint performance in trained men. J. Sports Med. Phys. Fit..

[13] Chatzopoulos D.E., Michailidis C.J., Giannakos A.K., Alexiou K.C., Patikas D.A., Antonopoulos C.B., Kotzamanidis C.M. (2007). Postactivation potentiation effects after heavy resistance exercise on running speed. J. Strength. Cond. Res..

[14] Turner A.P., Bellhouse S., Kilduff L.P., Russell M. (2015). Postactivation potentiation of sprint acceleration performance using plyometric exercise. J. Strength. Cond. Res..

[15] Cohen J. (1992). A power primer. Psychol. Bull..

